# Blood culture and antimicrobial susceptibility pattern of bacteria and fungi isolated from febrile neutropenic patients treated with chemotherapy at Taleghani hospital, Tehran

**Published:** 2019-04

**Authors:** Rozita Khodashahi, Mojdeh Hakemi-Vala, Masoud Mardani, Sara Abolghasemi, Ensieh Lotfali, Zahra Arab-Mazar, Naser Omidi, Sepideh Ghasemshahi

**Affiliations:** 1 Infectious Diseases and Tropical Medicine Research Center, Shahid Beheshti University of Medial Sciences, Tehran, Iran; 2 Department of Microbiology, School of Medicine, Shahid Beheshti University of Medical Sciences, Tehran, Iran; 3 Department of Medical Parasitology and Mycology, School of Medicine, Shahid Beheshti University of Medical Sciences, Tehran, Iran; 4 Section of Microbiology, Payvand's Clinical and Special Laboratory, Tehran, Iran

**Keywords:** Febrile neutropenia, Blood culture, Chemotherapy, Fungemia, Cancer

## Abstract

**Background and Objectives:**

The aim of this study was to determine the drug susceptibility pattern of the pathogens causing bacteraemia and fungemia in patients who have developed febrile neutropenia after chemotherapy.

**Materials and Methods:**

A total of 95 patients with suspected or proven malignancy (50 patients) were admitted to the adult haematology ward at Taleghani Hospital in Tehran. Blood samples were inoculated into the bottles of Bact/Alert blood culture system and sent to Payvand's clinical and special laboratory immediately and then incubated at 35 ± 2°C. Culture from positive bottles were plated on appropriate media and incubated at 37°C and 30°C for bacterial and fungal isolation, respectively. A bacterial suspension with turbidity equal to 0.5 McFarland (1.5 × 10^8^ CFU/mL) was prepared and used for the Vitec2 system (biomerioux). Statistical analysis using independent Fisher's exact test was conducted and a p-value of < 0.05 was considered as significant.

**Results:**

Among 50 patients with approved malignancy, Acute Lymphoblastic Leukaemia (ALL) and Acute Myeloid Leukaemia (AML) were the most common underlying diseases. This study showed, 20% (n: 10) of febrile neutropenic episodes established positive blood culture. Of them, 3 were Gram-negative (30%) and 5 were-Gram-positive bacteria (50%) and 2 patients (20%) showed fungemia with *Fusarium* spp.

**Conclusion:**

It is crucial to know about the likely pathogens and their local antibiotic and antifungal sensitivity patterns. Such local findings will show if any modifications to treatment guidelines are necessary.

## INTRODUCTION

Patients with hematologic malignancies are predisposed to severe infections, particularly during treatment with chemotherapeutics. The resulting neutropenia is a major cause of morbidity and mortality in these patients. Neutropenic cancer patients have a high risk of infectious complications, depending on the extent and duration of neutropenia, as well as on additional cellular and/or humoral immunosuppression and disruption of skin and mucosal barriers. Before the advent of the antibiotic era, mortality rates in neutropenic patients with leukemia and Gram-negative infections were as high as 91% (1). Over the last three decades, there have been considerable changes in the epidemiology of pathogens causing bacteremia in patients with febrile neutropenia. In the 1970s, Gram-negative infections caused 60–70% of bacteremia in neutropenic patients; in the 1990s, the majority of bacteremia occurred due to Gram-positive cocci (2–4). This trend has been attributed to many factors: widespread use of quinolones as prophylaxis, the use of central venous catheters and severe mucositis as a result of chemotherapy (5, 6). Also, invasive fungal disease (IFD) represents a major complication in patients with hematologic malignancies. It is important to make a definite diagnosis due to high mortality rates (7). Immunocompromised patients are at greatest risk of invasive fusariosis. This infection has been associated with solid organ transplantation, hematological malignancy, and prolonged neutropenia, and typically has a poor prognosis, with mortality rates exceeding 50%. Disseminated disease is the most frequent and challenging clinical form of fusariosis in immunocompromised patients, accounting for approximately 70% of all cases of fusariosis in this population. Patients at risk for disseminated fusariosis include those with acute leukemia and prolonged and profound neutropenia and patients undergoing HSCT. The most frequent pattern of disseminated disease is a combination of cutaneous lesions and positive blood cultures, with or without involvement at other sites (sinuses, lungs, and others). The typical clinical presentation of a patient with prolonged and profound neutropenia is persistent febrile, dissemination of infection and characteristic skin lesions, with positive blood culture (8–10).

Skin involvement is the first clue in most cases of disseminated fusariosis and often occurs at an early stage of the disease (8). Thus, disseminated infection should be suspected if multiple lesions are observed upon examination of the patient's skin (10). Hence, the aim of this study was to determine local patterns and drug susceptibility of the pathogens causing bacteremia and fungemia in patients who have developed febrile neutropenia after chemotherapy.

## MATERIALS AND METHODS

Patients were included in this study, if they met all the following three inclusion criteria: (I) fever; defined as a single oral temperature of 38.3°C or an oral temperature of 38°C lasting one hour, (II) neutropenia, defined as a count of neutrophil by <500 cells/mm^3^ or a count of <1000 cells/mm^3^ with a predicted decrease to <500 cells/mm^3^ within the next 48–72 hours and (III) received chemotherapy prior to the episode of febrile neutropenia. Other definitions of fever and neutropenia represent severe neutropenia as count of absolute neutrophil with less than 500 cells/μL and profound neutropenia with less than 100 cells/μL. The period of neutropenia is considered protracted if it lasts for >7 days. Based on the mentioned criteria, the patients were selected at Taleghani Hospital, Tehran-Iran as a referral hospital for patients with malignancies during March–August 2018. In a six month period, 95 patients with malignancy were admitted in adults haematology ward of the mentioned hospital. Febrile neutropenic patients who were aged above 14 years old and had not received empirical antibiotic therapy were included in the study. A total of 50 febrile neutropenic patients were enrolled. Patients, who had fever and neutropenia as a result of their underlying disease, which had not received chemotherapy, were excluded.

Subjects were interviewed by a physician and the data was collected including information on age, gender, underlying malignancy, corticosteroid in chemotherapy agent, antifungal and antibacterial as prophylaxis, recent antibiotic used, central venous catheter (CVC), absolute neutrophil count (ANC), skin lesion, lung involvement and serum Galactomannan and also cell blood count (CBC) (Fig. 2). For all patients that fever and neutropenia continued after 4 days without a diagnosis of any source, detection of serum Galactomannan, Para Nasal Sinus (PNS), CT and Chest CT Scan were requested.

### Blood collection, CBC and Bact/ Alert system.

12 ml of the venous blood sample was obtained aseptically from each patient via venepuncture; according to a standard technique after skin disinfection.10 ml of collected blood was used for blood bottle inoculation. Inoculated blood bottles were incubated at 35 ± 2°C in the BacT/ALERT blood culture System (BacT/Alert FA Plus, bioMerieux SA, France). The BACT/Alert bottles that showed a sign of growth were alarmed. All samples were sent to Payvand's clinical and special laboratory, immediately and were incubated at 35 ± 2°C in Bact/Alert blood culture system. 2 ml of remained collected blood was added to CBC container including EDTA and was sent to the hospital lab.

### Bacterial identification and antimicrobial susceptibility testing (AST) using Vitec 2 system.

Pure bacterial cultures are needed to work with this system. So, all positively alarmed bottles of Bact/ Alert system were sub-cultured on 5% sheep blood culture, chocolate agar and Sabouraud dextrose agar (SDA) were incubated at 37°C and 30°C for bacterial and fungal growth, respectively.

Bacterial identification and antibiotic susceptibility testing (AST) was done using automated Vitec 2 system (bioMerieux, France). In order to choose suitable identification (ID) and AST cards, basic identification tests such as Gram staining, catalase and oxidase tests were done according to standard bacteriology protocol on all positive blood agar and chocolate agar plates.

Based on Vitec2 construction, bacterial suspension was prepared from each pure culture with turbidity equal to 0.5 McFarland (1.5 × 10^8^ CFU/ml). All prepared bacterial suspensions and special ID and AST cards were inserted into vitec2 system apparatus, simultaneously. AST was determined after identification by determining the minimum inhibitory concentration (MIC) for each antibiotic. All antibiotics were selected based on CLSI guideline (11).

### Fungi identification and antifungal susceptibility testing (AST).

Despite the possibility of identification of *Candida* spp. and its antifungal susceptibility test (AST) using Vitec2 system, mold's or fleshy fungi identification and their AST were determined manually. So, for all SDA plates which revealed fungus colonies, the microscopic evaluation was done by methylene blue. Mould's identification was done based on standard mycology tests.

Antifungal agents including; amphotericin B (MIC range 0.5–2 μg/mL) (Sigma-Aldrich, USA), Voriconazole (MIC range 0.031–16 μg/mL) (Pfizer Central Research, UK), and Caspofungin (Merck, USA) were used in AST.

## RESULTS

### Patients profile.

**Fig. 1.**
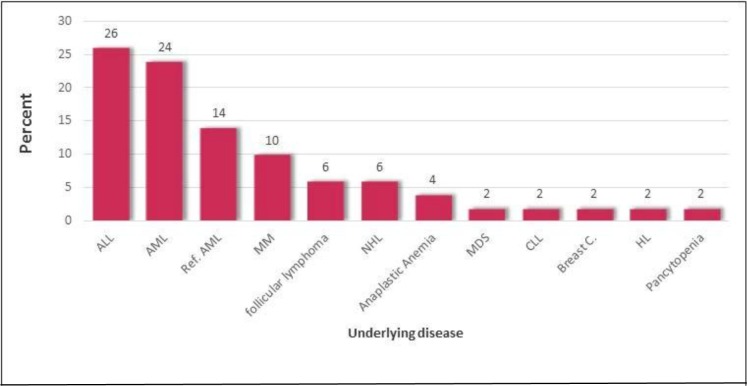
Underlying cancer diseases ALL: Acute lymphoblastic leukemia. AML: Acute myeloid leukemia. MM: Multiple Myeloma. NHL: Non Hodgkin lymphoma. MDS: Myelo dysplastic syndrome. CLL: Chronic lymphoblastic leukemia. Breast c: Breast cancer. HL: Hodgkin lymphoma

**Fig. 2.**
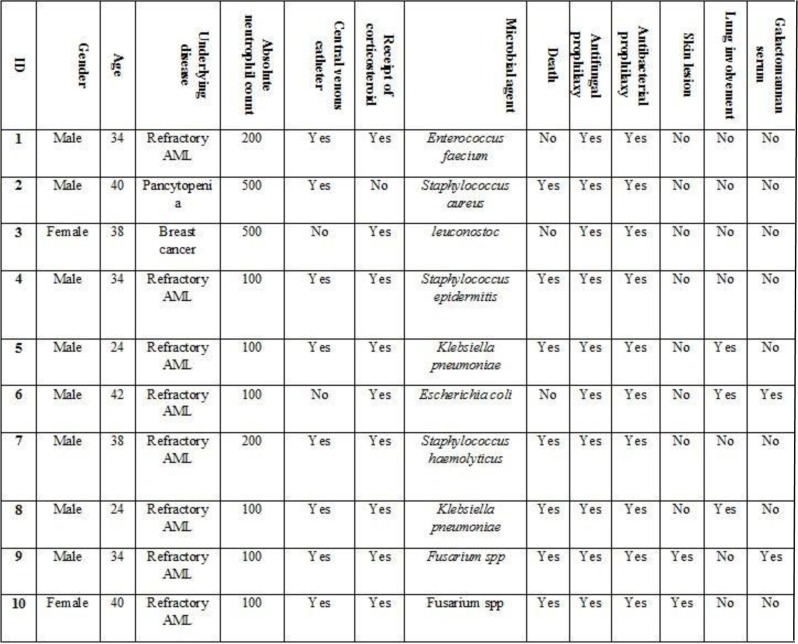
Demographic and patients information after physician review

The median age of patients studied at Taleghani hospital of Tehran was equal to 42.6 years (±ranging 24–65 years). The ratio of males to females was almost 5:1 during April–August 2018. The majority of patients suffered from acute lymphoblastic leukemia (26%) and acute myeloid leukemia (24%) (Figs. 1 and 2).

All of the patients had received chemotherapy. The median range of ANC was equal to 100–700 cell/μL (352 ± 184.32). In this study, 36% (n=18) of patients had a central venous catheter. All patients with refractory AML received mitoxantrone, etoposide, and cytarabine (EMA) regimen.

Also, 78% (39) of patients had received corticosteroid in their chemotherapy regimen. The 50 episodes of febrile neutropenia were studied based on the criteria mentioned before.

### Identification of microorganisms.

All of these episodes showed a positive culture with a single pathogen. The identified agents isolated from patients were Gram-negative bacteria (n=3, 30%), Gram-positive bacteria (n=5, 50%) and fungi (n=2, 20%).

Among Gram-positive bacteria, *Enterococcus faecium, Staphylococcus aureus, Staphylococcus epidermidis* and *Staphylococcus haemolyticus* were identified using Vitec2 Gram-positive ID card. Among Gram negative isolated bacteria, *E. coli* and *Klebsiella pneumoniae* were detected using Vitec2 Gram-negative ID card. All Gram-negative isolates were oxidase negative. All *Staphylococcus* spp. showed positive in catalase test and all *Enterococcus* spp were catalase negative.

### Antimicrobial and antifungal sensitivity patterns.

**Fig. 3.**
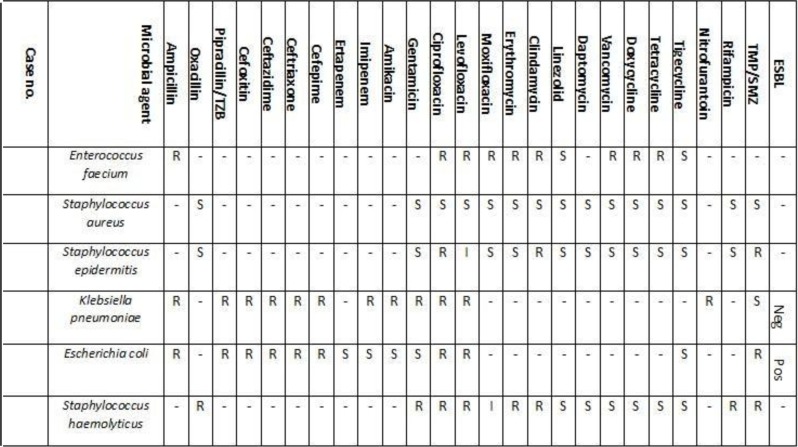
Antibiotic susceptibilities profile of each bacteria isolates from febrile neutroprnic patients TZB: Tazobactam, TMP/SMZ: Trimethoprim/Sulfamethoxazole, ESBL: extended-spectrum beta-lactamases, R: resistant, S: sensitive, I: intermediate, Neg: negative, Pos: positive

Details of the *in vitro* sensitivity profiles of isolated bacteria are shown in Fig. 3. Gram-negative isolates including two isolates of *K. pneumoniae* were resistant to imipenem and were sensitive to tigecycline, and one strain of *E. coli* was identified as ESBL producer using the Vitec-2 system. *E. faecium* was only sensitive to linezolid and tigecycline. All *Staphylococcus* spp isolates were sensitive to vancomycin. The isolates of *S. aureus* were identified as methicillin-sensitive based on the results of using cefoxitin and Vitec-2 systems report (Fig. 3).

Two plates of SDA showed mold-like colonies. In microscopic evaluation, the production of hyaline was detected as banana multicellular macroconidia with foot cell at the base. Based on the microscopic properties, presumptive identification was *Fusarium* spp. and later was confirmed by PCR and sequencing (12), but the the method and sequencing result is not indicated here. Antifungal susceptibility test was performed using the microdilution method according to CLSI document M38-A2 (13). In the antifungal microdilution test, antifungal concentration was the endpoint that produced complete inhibition of visual growth at 48 h. The MIC endpoint for the voriconazole and amphotericin B was defined as the lowest concentration that produced complete inhibition of growth, whereas the minimum effective concentration (MEC) endpoint for caspofungin was defined according to previous researches (13, 14).

In this study, *Fusarium* spp. was susceptible to amphotericin B, voriconazole and caspofungin. No, any species of *Candida* was detected using Vitec -2 system.

## DISCUSSION

In order to treat effectively infections in the neutropenic patient with fever, it is crucial to know about the likely pathogens and the local antibiotic and antifungal sensitivity patterns in individual centres. In a multicentre study on febrile neutropenic patients in the USA, Wisplinghoff et al. reported the outweigh of Gram-positive microorganisms as a cause of 62% and 76% of blood stream infections in 1995 and 2000 respectively, while only 22% and 14% of all blood stream infections were originated from Gram-negative ones at the same time (15). Another report from a New Zealand hospital also showed the predominance of Gram-positive bacteria. In this study, Gram-positive cocci accounted for 46% of isolates while only 35% of isolates were Gram-negative bacilli (16). Castagnola et al. in their study reported that 57% and 41% of bacteremia in febrile chemotherapy-induced neutropenia were caused by gram-positive and negative organisms respectively (17). In this study, the etiology of bacteremia in febrile neutropenic patients was showed Gram-positive bacteria 55.5% predominantly after administrating the chemotherapy. This pattern has been explained by some factors, such as, increased incidence of severe mucositis as one of the important factors resulting from increasingly potent chemotherapy, widespread use of quinolones as prophylaxis which are more active against the Gram-negative bacteria and frequent use of central venous catheters which contributes to developing skin-derived Gram-positive infections (18). Hand washing, better isolation and generally better sanitation of patients in oncology wards were other reasons causing this event (19).

Based on the results, 10 of 50 episodes (20%) were blood culture positive using Bact/ Alert blood culture system. Except for one case with leuconostic bacteremia that complained as contamination, all of the patients with Gram-positive bacteremia and patient with fungemia had a central venous catheter (CVC). Except for one case of leuconostic bacteremia that complained as contamination and one patient with pancytopenia without a definite diagnosis, another bacteremia and fungemia occurred in refractory of AML patients who received mitoxantrone, etoposide, and cytarabine (EMA) regimen. There were no CVC in patients with Gram-negative bacteremia.

All staphylococci isolates were sensitive to vancomycin. Thus, vancomycin could still be the choice for empirical treatment when staphylococci are suspected. We had significantly higher rates of bacteremia in acute myeloid leukemia patients in this study. This is likely due to the more myelosuppressive chemotherapy, which usually results in a longer duration of neutropenia, as a known risk factor for developing infections (20). Profound neutropenia is also a risk factor for developing an infection such as ESBL-producers, carbapenem-resistant Enterobacteriaceae and *Fusarium* spp.

Surveillance is also important for monitoring the rates of resistant organisms, such as ESBL-producers and carbapenem-resistant Enterobacteriaceae. Immunocompromised patients are at the greatest risk of invasive fusariosis. This infection has been associated with solid organ transplantation, hematological malignancy, and prolonged neutropenia, and typically has a poor prognosis, with mortality rates exceeding 50% (8–10).

Skin involvement is the first clue in most cases of disseminated fusariosis and often occurs at an early stage of the disease. Multiple erythematous macular or popular painful lesions are reported in 70% of cases. Lesions usually have a necrotic center resembling ecthyma gangrenosum and are also described as ecthyma gangrenosum-like lesions (10). In patients with hematological malignancies and prolonged fever and neutropenia, it is important to make a differential diagnosis between fusariosis and invasive pulmonary aspergillosis due to their similar clinical presentations. The negativity of galactomannan antigen and the absence of typical radiological signs of aspergillosis can favor a diagnosis of fusariosis, whereas recent studies reported that, galactomannan can also be positive in cases of fusariosis (9, 10). In this study, serum galactomannan was positive in only one case of invasive fusariosis, and skin lesion and positive blood culture occurred exclusively in a patient with IF (invasive fusariosis). Thus, disseminated infection should be suspected if multiple lesions are observed upon examination of the patient's skin (10). Treatment of disseminated fusariosis can be difficult and despite its susceptibility to all tested antifungal agents in the recent study, we did not have neutrophil recovery and it was a reason for poor outcome. Fungemia and bacteremia with Gram-negative bacteria were occurred in profound neutropenia (ANC: 100).

**Fig. 4.**
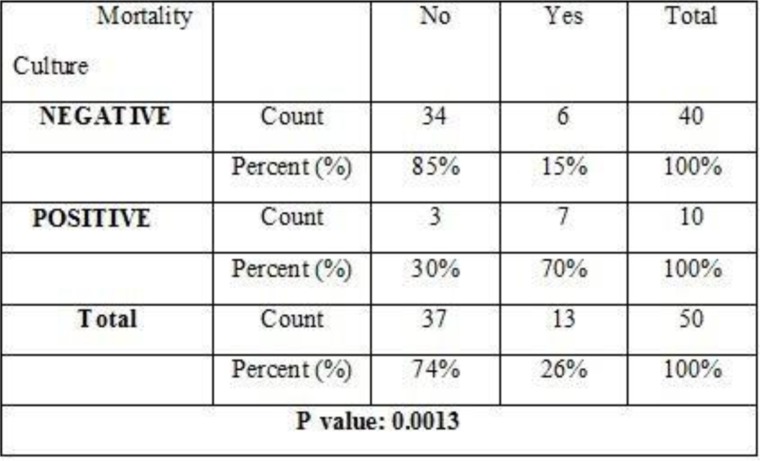
Mortality rate related with blood culture

In follow up approach until the last day of admission revealed that 26% (n=13) of patients died. Of them, 70% of patients were positive blood culture and 15% of patients were negative blood culture. These data were revealed according to the results of Fisher's Exact test (p=0.0013) (Fig. 4).

Skin lesion and positive blood culture occurred exclusively in a patient with IF (Invasive Fusariosis). Also, lung involvement occurred only in resistance Gram-negative bacteremia, such as ESBL-producers and carbapenem-resistant Enterobacteriaceae, and also serum Galactomannan was positive in only one case of invasive fusariosis and resistant organisms, such as ESBL-producers. All of the Gram-negative bacteremia (three cases) was proven regarding invasive pulmonary aspergillosis (IPA) as well as bacteremia.

In patients who have febrile neutropenia after chemotherapy, it is important for the attending clinicians to risk-stratify the patients in order to prevent any fatal complications.
